# Delirium and Psychiatric Sequelae Associated to SARS-CoV-2 in Asymptomatic Patients With Psychiatric History and Mild Cognitive Impairment as Risk Factors: Three Case Reports

**DOI:** 10.3389/fpsyt.2022.868286

**Published:** 2022-04-07

**Authors:** Michele Fabrazzo, Antonio Russo, Mario Luciano, Alessio Camerlengo, Pierluigi Catapano, Bianca Amoroso, Francesco Catapano, Nicola Coppola

**Affiliations:** ^1^Department of Psychiatry, University of Campania “Luigi Vanvitelli”, Naples, Italy; ^2^Infectious Diseases Unit, Department of Mental Health and Public Medicine, University of Campania “Luigi Vanvitelli”, Naples, Italy

**Keywords:** SARS-CoV-2, COVID-19, delirium, mild cognitive impairment, psychiatric sequelae, neuroinvasion, disorganized/mutacic speech time/space disorientation, reference/persecutory delusions

## Abstract

Human coronaviruses have neuroinvasive and neurotropic abilities that might explain psychiatric outcomes in affected patients. We hypothesized that delirium might be the sole clinical manifestation or even the prodrome of a psychiatric episode consistent with the mental history of a few infected patients with a preexisting diagnosed cognitive impairment. We examined three patients with preexisting mild cognitive impairment and delirium at admission for suspected SARS-CoV-2 infection. We diagnosed delirium using DSM-5 and Confusion Assessment Method (CAM) and measured consciousness level by the Glasgow Coma Scale. All the patients had no history of fever, respiratory complications, anosmia or ageusia, meningitis, and negative cerebrospinal fluid analysis for SARS-CoV-2. Our first patient had no psychiatric history, the second reported only a depressive episode, and the third had a history of bipolar disorder dated back to 40 years before. In the first patient, delirium resolved 2 days following the admission. The other two patients recovered in 4 and 14 days, and delirium appeared as the prodrome of a new psychiatric episode resembling past events. Clinicians should monitor the possibility that SARS-CoV-2 presence in the brain might clinically manifest in the form of delirium and acute psychiatric sequelae, even without other systemic symptoms. Psychiatric history and preexisting mild cognitive impairment are to be considered as predisposing factors for COVID-19 sequelae in delirium patients.

## Introduction

COVID-19 is a disease caused by the severe acute respiratory syndrome coronavirus 2 (SARS- CoV-2) and represents a challenge to the world health care system (1).

The disease may be clinically associated with a large spectrum of physical and mental health manifestations, heavily affecting the well-being of the worldwide population (2–4).

Atypical presentations of SARS-CoV-2 infection have appeared since the pandemic outbreak in 2019 and include gastrointestinal symptoms, multiorgan failure (liver, kidneys, heart), neurologic and psychiatric manifestations (5–8). In particular, patients with COVID-19 may present psychiatric manifestations such as depressive and anxiety symptoms (9), increased risk of suicide (10, 11), and insomnia (8). Furthermore, delirium appears as one of the most frequent conditions in emergency settings among psychiatric consequences of COVID-19 infection. Therefore, delirium is generally considered an indicator of brain vulnerability and predisposed by factors such as older age, presence of comorbid medical illnesses, dementia, or preexisting cognitive impairment. However, at the moment, the mechanisms underlying the association between COVID-19 and delirium remain unknown, though existing evidence suggests a multifactorial etiology (12).

Worldwide, scientists are still debating whether neurologic and psychiatric manifestations originate from SARS-CoV-2’s impact on the brain or result from the psychological distress related to the infection and the pandemic (13). Most studies focused on the general population’s psychological impact of COVID-19 (14, 15) during the current pandemic, while less attention was devoted to the more vulnerable population affected by mental illness (9, 16).

In particular, the literature has not widely scrutinized the association between mental illness and risk of SARS-CoV-2 infection yet. Therefore, evidence remains scarce on whether people with a mental health condition might show different susceptibility to virus infection or worse clinical outcomes (17). Also, further factors might have contributed to triggering or aggravating preexisting mental illnesses (i.e., isolation, loneliness, socioeconomic and poor health conditions, high levels of perceived stress) (18, 19). Furthermore, the limited access to mental health facilities during the COVID-19 pandemic might have an indirect effect on psychiatric disorders, exacerbating or increasing those concerning patients with poor treatment response and reduced probability to recover with standard treatments (8, 20, 21), plus those related to substance use (7, 22, 23). Scientists expressed concerns about the subjects diagnosed with a mental disorder being at a higher risk of developing COVID-19 with a worse outcome (24). Nevertheless, scientific evidence on psychiatric diagnosis as a potential risk factor for severe or fatal COVID-19 is still limited (25, 26).

To this aim, Lee et al. (18) reported that a preexisting mental disorder diagnosis was not associated with an increased probability of testing positive for SARS-CoV-2.

The authors highlighted that patients with a severe mental illness had a slightly higher risk for severe clinical outcomes of COVID-19 than patients without a history of mental illness. In addition, patients with non-affective or affective disorders with psychotic features had a slightly greater risk of severe clinical outcomes of COVID-19 than patients with other mental illnesses or without a mental illness. On the other hand, Taquet et al. (27) investigated whether patients with COVID-19 showed an increased rate of psychiatric disorders diagnosed after the virus infection onset. In addition, the authors examined whether patients with a history of psychiatric illness were especially at higher risk of receiving a diagnosis of COVID-19. Data from this retrospective cohort study emphasized that COVID-19 survivors appeared to be at increased risk for psychiatric sequelae and that a psychiatric diagnosis might be an independent risk factor for such an infectious disease. On the other hand, patients diagnosed with COVID-19 without a psychiatric history showed an increased incidence of a first psychiatric diagnosis in the 14–90 days after the diagnosis of infection and a higher risk of experiencing anxiety disorders, insomnia, and dementia.

Pandharipande et al. (28) reported that survivors of critical illness presenting with delirium during hospitalization, frequently show a prolonged cognitive impairment which is characterized by deficits, newly appeared or exacerbations of preexisting mild deficits in global cognition or executive functions. Pinna et al. (29) reported that 24% of a cohort of patients diagnosed with COVID-19, had cognitive abnormalities (mostly short-term memory loss), but did not specify whether they were prior or resulted as a complication of the viral infection. On the other hand, Helmes et al. (30) mentioned that, in a SARS-CoV-2 infection cohort, 33% of patients (15/45) had a dysexecutive syndrome consisting of inattention, disorientation, or poorly organized movements in response to commands after discharge. In addition, Becker et al. (31) and Graham et al. (32) reported that COVID-19 survivors frequently suffered from cognitive dysfunction, described commonly as “brain fog”, greatly affecting their quality of life.

SARS-CoV-2 infection is associated with an increased risk of long-term cognitive decline especially in the elderly population (33). Furthermore, several chronic medical conditions affecting older adults might further increase the risk of developing COVID-19 and deteriorating cognitive functions (34).

Alzheimer’s disease and related dementias are other age-related chronic diseases (35). Several risk factors for COVID-19 overlap with those for dementia, suggesting that older adults with cognitive impairment might be particularly susceptible to COVID-19 (36). Besides risk factors, dementias and COVID-19 might share comorbidities such as hypertension, diabetes, and obesity, most of which are associated with an overactive renin-angiotensin system, cerebrovascular dysfunction and neuroinflammation (12). The study of Pisaturo et al. (37) reveals that patients with a previous diagnosis of dementia were more vulnerable than matched control patients without dementia, plus at increased risk of severe COVID-19 and consequent death.

It is still to be clarified whether a preexisting mild cognitive impairment represents a risk factor for short and/or long-term complications of SARS-CoV-2. Pan et al. (38) reported that preexisting cognitive impairment was significantly associated with increased susceptibility to SARS-CoV-2. Finally, the authors highlighted that cognitively impaired individuals were older and showed a higher cardiovascular and cerebrovascular comorbidity burden.

Human coronaviruses have neuroinvasive and neurotropic abilities that might explain delirium and/or other psychiatric disorders’ onset, outcomes, and cognitive impairments (33). Indeed, several reports highlighted the increasing association between COVID-19 and psychiatric manifestations, including delirium, sleep disorders, anxiety, and depression. In particular, the clinical onset of impaired consciousness and/or delirium in most cases suggest that the virus penetrates the brain and spreads to the neocortex (39).

We aimed to examine whether delirium, in particular, might be the sole manifestation of SARS-CoV-2 brain infection without systemic or multiorgan failure in patients without a psychiatric history. Also, we speculated that the mental manifestations of COVID-19, initially presenting as a confusional state, might produce a different outcome in patients who experienced psychiatric episodes in the past.

To this aim, we described three clinical cases of patients with delirium as the outburst of SARS-CoV-2 infection that resolved or appeared as the prodrome of a new psychiatric episode mimicking those patients had experienced in the past.

The clinical relevance of mild cognitive impairment of each patient prompted us to formulate a further hypothesis based on cognitive impairment as a predisposing factor for COVID-19.

## Case Presentation

We analyzed three clinical cases of patients diagnosed with delirium according to the Diagnostic and Statistical Manual of Mental Disorders 5 (DMS-5) criteria (40).

In addition, we used the Confusion Assessment Method (CAM) (41), which includes four cardinal features of delirium: (1) acute onset and fluctuating course, (2) inattention, (3) disorganized thinking, and (4) altered level of consciousness. Specifically, a diagnosis of delirium applying the CAM requires the presence of at least features 1 and 2, and either 3 or 4. Furthermore, we administered the Glasgow Coma Scale (GCS) to measure patients’ level of consciousness based on their ability to perform eye movements, speak, and move their bodies (42). The diagnosis of mild cognitive impairment was made retrospectively based on the anamnesis provided by patients’ family members. Indeed, a few years earlier, our three patients, due to memory and thinking complaints, had undergone a neurological examination and performed a Mini-Mental State Examination (MMSE), obtaining a score <24, as illustrated in Table 1. The MMSE (43) is a test used to assess the severity and progression of cognitive impairment and includes questions in different areas, grouped into seven categories, each representing a different domain or function: (i) orientation to time; (ii) orientation to place; (iii) registration of three words; (iv) attention and calculation; (v) repetition of the three words; (vi) language construction, (vii) visual construction. A score of 23 or less indicates cognitive impairment, whose severity is classified as follows: 24–30 = no cognitive impairment; 18–23 = mild cognitive impairment; and 0–17 = severe cognitive impairment.

**TABLE 1:** Medical and psychiatric characteristics of the three patients included in the analysis.

	Patient 1	Patient 2	Patient 3
Medical comorbidities	Paroxysmal atrial fibrillation, hypertension, thyroid nodules	Essential hypertension	Previous pulmonary and external iliac artery embolism; decomposed fracture of the right homer; amputation of the left leg
Psychiatric history	None	A single depressive episode Long-term treatment with prazepam	Bipolar disorder Long-term treatment with antipsychotics, mood stabilizers, and benzodiazepines
Preexisting MCI diagnosis based on:	Neuropsychological test (MMSE score:22)	Neuropsychological test (MMSE score:23)	Neuropsychological test (MMSE score:22)
Admission medical parameters			
*Temperature*	35.6°C	36.7°C	37.5°C
*Blood pressure (mmHg)*	159/59	115/60	100/65
*Heart rate (beats/minute)*	101	73	71
*Respiratory rate*	20	18	22
*Oxygen saturation, room air*	97%	100%	95%
*SARS-CoV-2 test*	Positive	Positive	Positive
*SARS-CoV-2 RNA in CSF*	Not performed	Negative	Not performed
*Glasgow coma scale (GCS) score*	11 (subscores 4, 2, 5)	15 (subscores 4, 5, 6)	12 (subscores 4, 4, 4)
Confusion assessment method (CAM)	3/4	3/4	3/4
Clinical features: -*at COVID-19 onset*	Altered and fluctuating level of consciousness	Chills, stiffening, transitory absence, clonic contractions of the right upper limb	Insomnia, agitated behavior
*-at admission*	Disorganized/mutacic speech, disorientation	Time/space disorientation, psychomotor agitation, speech difficulty, guilty delusions	Reference/persecutory delusions
*-at discharge*	Complete recovery after 2 days of treatment with promazine and dexmedetomidine	Complete recovery after 4 days of low-dose haloperidol (0.5 mg/t.i.d.)	Complete recovery after 2 weeks of antipsychotic and mood stabilizer treatment
Overall psychiatric symptoms	None	Depressed mood, apathy, mutism, crying, guilty delusional beliefs	Auditory hallucinations, delusional beliefs, pressured speech, manic symptoms, emotional liability
Length of patient’s hospitalization	7 days	7 days	12 days

*MCI = mild cognitive impairment; MMSE = Mini-Mental State Examination.*

During the hospitalization, the neurological examination of the three patients revealed no weakness in the upper and lower limbs, no prevailing decubitus, and no signs of meningitis. All patients’ cranial nerves were intact, and no neck stiffness, photophobia, or focal neurological deficits emerged. Brudzinski’s and Kernig’s signs and other indicators of meningitis were negative. The flexor plantar response was normal. Findings of cardiovascular and abdominal examinations were irrelevant.

No evidence of acute or subacute brain insult emerged after performing the nasopharyngeal swab and a computerized tomography (CT) of the skull.

Even after repeating the CT scan after 48 h, the same result was confirmed. Additionally, no patients manifested fever, shortness of breath, sore throat, gastrointestinal symptoms, anosmia, or ageusia. Finally, a septic blood screen and urine culture tested negative.

Table 2 outlines the demographic characteristics and laboratory admission parameters of the three patients who received nasopharyngeal swab testing for SARS-CoV-2 following recent close contact with infected subjects. Table 1 summarizes the clinical features at COVID-19 onset, admission, and discharge of the three patients.

**TABLE 2:** Demographic characteristics and laboratory parameters at admission of the three patients included in the study.

Demographic variables	Patient 1	Patient 2	Patient 3
*Age*	68	71	72
*Gender*	F	F	M
*Laboratory parameters*			
*Red blood cells* × *10^6^/*μ *L*	4.84	4.83	3.92
*White blood cells* × *10^3^/*μ *L*	10.9	7.43	3.87
*Hemoglobin, g/dL*	12.9	13.6	12.5
*Platelets* × *10^3^/*μ *L*	371	194	109
*Neutrophils* × *10^3^/*μ *L*	8.60	4.75	2.16
*Lymphocytes* × *10^3^/*μ *L*	1.30	2.07	1.47
*Monocytes* × *10^3^/*μ *L*	1.00	0.61	0.24
*Na* + , *mEq/L*	148	138	138
*C-reactive protein (CRP), mg/L*	28.8	3.0	10.7

The first patient had no psychiatric history; the second only a depressive episode occurred more than 30 years before; the third a history of bipolar disorder dated back to almost 40 years before.

The first patient remained asymptomatic until mid-December 2020 when she suddenly woke up presenting an altered level of consciousness at night. Her relatives described her standing in the kitchen, mutacic and without control of her sphincters. Therefore, she was transported to the hospital. She continued to be mutacic in the emergency room, though she alternated agitated behavior and disorientation to time, space, and people. Finally, she was hospitalized and treated with promazine intramuscular (25 mg/ml), administered when she was agitated, or dexmedetomidine by intravenous injection in 50 ml of saline solution and a flow of 2 ml/h to provide sedation with no risk of respiratory depression. The daily treatment also included pantoprazole (40 mg/day) and acyclovir (250 mg t.i.d.). The patient recovered after 2 days of treatment with promazine and dexmedetomidine.

The second patient, at the beginning of November 2020, a few days after her husband’s death, presented chills, stiffening, and impairment of consciousness associated with clonic contractions of the right upper limb.

Before reaching the emergency unit, she reported an episode of atrial fibrillation. Two nasopharyngeal swabs for SARS-CoV-2 previous to hospitalization tested negative. The patient repeated the test in the emergency room and received a positive result. The CT of the skull showed only preexisting hypoperfusion of the periventricular white matter. Furthermore, she received a lumbar puncture (LP) to test for SARS-CoV-2 central nervous system (CNS) involvement. The patient did not develop active respiratory symptoms or require oxygen therapy.

At admission to our COVID-19 center, she appeared agitated, confused, with disorganized thinking, and disoriented to time, space and people. The following days, she described herself as being deceased, hopeless, complaining against medical staff for being “unable to save her.” She also felt ashamed for “doing something wrong” and doing nothing to avoid it. She repeatedly stood on the bed, running the risk of falling. Furthermore, she alternated mutism with senseless stuttering and speech difficulty. The patient had already experienced a depressive episode with delusional ideas, treated for more than 30 years exclusively with a benzodiazepine, recently at increased doses due to emerging anxiety due to her husband’s death. After the psychiatric consultation, she was treated with intramuscular promazine (50 mg/t.i.d.) and diazepam e.v. (10 mg) in case of agitated behavior. The day after, the consultant psychiatrist prescribed sodium valproate (1000 mg/day) and haloperidol (1.5 mg/day), along with enoxaparin sodium (4000 UI/day). Unlike the first patient, she required 4 days of continuous treatment with sodium valproate and low doses of haloperidol to recover.

The third patient was admitted to our psychiatry ward and then transferred to the COVID center of our university. At the end of September 2020, he experienced psychomotor agitation, confusion, disorganized thinking, and disorientation to people. In addition, persecutory, reference, and poisoning delusions appeared in the following days, leading to non-compliance to treatment.

Due to close contact with his physiotherapist, who tested positive for COVID-19, he was isolated at admission and then transferred to the Infectious Diseases Unit (IDU) after testing positive for SARS-CoV-2 upon nasopharyngeal swab. When admitted to the COVID-19 unit, the patient was on psychopharmacological therapy, including a mood stabilizer (sodium valproate, 1000 mg/day), a first- (haloperidol, 3 mg/day), and a second-generation antipsychotic (olanzapine, 10 mg/day), plus promazine (30 mg as needed). A few days later, doses were increased as follows: sodium valproate to 1500 mg/day, haloperidol to 4 mg/day, and olanzapine to 20 mg/day due to agitated behavior and persisting delusional ideas. Furthermore, promazine (50 mg) was administered i.m. at bedtime because of insomnia. The patient required 2 weeks of antipsychotic and mood stabilizer treatment to be stabilized.

No patients manifested medical or psychiatric sequelae of COVID-19 or delirium during the follow-up after discharge.

## Discussion

We described three clinical cases of patients presenting delirium as an initial outburst of SARS-CoV-2 infection. Such mental state change did not persist in one patient and appeared as the prodrome of a psychiatric episode in the remaining two patients. Furthermore, a feature common to all three patients was the preexisting mild cognitive impairment, though diagnosed a few years before SARS-CoV-2 infection and based only on anamnestic information for two patients. Delirium was the sole clinical manifestation of SARS-CoV-2 brain infection in one patient, with no additional systemic or multiple organ failure symptoms. We also reported that delirium manifested as a confusional state and behavioral manifestations in the remaining two patients, then it evolved mimicking past psychiatric episodes.

Whether SARS-CoV-2 infection appears as a delirium condition also in patients with a mild preexisting cognitive impairment is still controversial. However, a few studies have reported on the comorbidity of SARS-CoV-2 infection and delirium in patients with concurrent cognitive impairment in adults ≥18 years. Cognitive impairment is described in several case reports and case series of patients presenting delirium or delirium-like symptoms as the main outburst of COVID-19 (44, 45). Indeed, in most cases, a mild-to-severe cognitive impairment was evidenced before, during, or after the acute phase of the infection.

Some studies described the analyzed patients as having a past neurologic or psychiatric history (46–48). In line with our results, Zhou et al. (48) indicated that delirium, among all the disorders characterized by cognitive dysfunctions, appeared to be a predictor for developing other neuropsychiatric events during hospitalization.

Instead, contrasting data arose regarding the potential relationship between COVID-19 and delirium in patients with or without premorbid cognitive impairment and without a past psychiatric history. In contrast to what we observed in our first patient, Parker et al. (49), along with Wittock and Van Den Bossche (50), reported psychotic symptoms after delirium in patients with premorbid cognitive impairment and no psychiatric history. Our three patients share the only feature of the preexisting mild cognitive impairment that might have acted as a predisposing factor for COVID-19-related delirium. Instead, psychiatric history and cognitive impairment might have played the role of predisposing factors for COVID-19 sequelae in delirium patients. However, it remains demanding to ascertain whether our patients’ psychiatric manifestations, following the initial outburst of delirium, represented a prolongation of delirium itself or symptoms of a new psychiatric episode onset mimicking those that occurred in our patients in the past. Thus, a casual relationship between COVID-19-related delirium and psychiatric symptoms cannot be inferred entirely. Indeed, delirium features itself as a widely heterogeneous condition and psychiatric sequelae may represent part of its clinical presentation. Furthermore, we cannot exclude that our patients’ psychiatric manifestations were due to the psychological response to COVID-19.

Indeed, the death of our second patient’s husband might be identified as a triggering event influencing the outcome of the infectious disease.

We included three clinical cases of older adults (age >65 years) presenting at our hospital with an unusual manifestation of COVID-19, namely an agitated confusional state without respiratory symptoms or fever. Therefore, recognizing delirium as a clinical manifestation of COVID-19 in our patients appeared crucial for achieving an accurate diagnosis and providing suitable care to prevent outbreaks in the healthcare facilities during the pandemic.

Criteria on which a diagnosis of delirium is formulated encompass the rapid onset of symptoms, quick recovery, amnesia, and predominance of confusion throughout the event (40). Indeed, our first patient presented a premorbid decline in functioning and memory, as observed by her relatives, no psychiatric history, and a delirium condition as the sole manifestation of COVID-19. Our second and third patients also had a diagnosed premorbid mild cognitive impairment and a delirium state manifesting at the COVID-19 onset. In addition, they both afterward experienced a psychiatric episode resembling the episodes that occurred in the past. Furthermore, our second patient manifested a depressive state with several mood-congruent features such as depressed mood, apathy, mutism, crying, and guilty delusional beliefs. Finally, our third patient showed manic and psychotic symptoms (Table 1).

Our study aligns with part of the scientific literature (14, 26, 51, 52), which reveals that SARS-CoV-2 infection might cause delirium in a large number of patients in the acute phase of the infection and that patients with a preexisting mental disorder might be at increased risk for COVID-19. However, little is yet known about SARS-CoV-2 patients presenting delirium in the absence of a psychiatric history.

Taquet et al. (27) reported on this topic that infected patients without a psychiatric history showed an increased incidence of a first psychiatric diagnosis in the 14–90 days following the onset of the infectious disease. Contrarily, our first patient presented no complications after recovery (Table 1).

Our cohort includes a heterogeneous set of individuals with an atypical presentation of Covid-19, which appeared as a changed mental status as the primary sign of infection, requiring emergency room care.

Table 2 shows that 2/3 of our patients are females and >65 years, and all had a mild cognitive impairment previously diagnosed.

Instead, no patients presented associated cerebrovascular ruptures involving the brain parenchyma or the subarachnoid space, peripheral nerves, neuromuscular junctions, skeletal muscles, and other medical conditions, as illustrated in a few studies (5, 53).

Differently, Mazza et al. (51) reported that cognitive impairment (dysfunctions in attention and information processing) was strictly related to depressive symptoms in the 3 months following the viral infection and systemic inflammation. In addition, the authors observed no considerable difference in cognitive performance tests between patients with and without a psychiatric history, in line with our findings.

It should be noted that all our patients shared the absence of the core features of COVID-19, such as prominent respiratory and gastrointestinal symptoms. Nevertheless, moderately elevated inflammatory markers, especially C-reactive protein (CRP), might suggest a dysregulated immune response as a possible delirium precipitant.

The pathophysiology by which SARS-CoV-2 causes CNS involvement is yet to be elucidated. However, the severity of systemic illness (and the associated metabolic derangements besides inflammatory cascades) in some patients with COVID-19 is probably sufficient to cause the toxic-metabolic encephalopathy frequently present in hospitalized patients (5). Furthermore, in line with what reported by Masi et al. (54), COVID-19 is associated with a systemic coagulopathy favoring thromboembolic complications and thromboinflammation. In this perspective, systemic inflammatory response syndrome might be a major contributor to COVID-19 associated coagulopathy. In addition, a septic condition might represent a deleterious systemic inflammatory response to infection. Lin et al. (55) hypothesized that plasma metabolism could clarify sepsis complicated by organ dysfunction, as it may probably occur in COVID-19 patients.

Nevertheless, atypical COVID-19 appearance in patients with severe confusional states without respiratory symptoms or other organ failures suggests considering alternative mechanisms by which the virus might cause mental alterations, including the hematogenous SARS-CoV-2 spread to the CNS through the periventricular system (56).

Furthermore, delirium and frontal-lobe-related behavioral changes are repeatedly present in COVID-19 patients. In particular, these symptoms appear as associated with motor signs, such as corticospinal signs, myoclonus, rigidity, and akinetic mutism (57, 58). COVID-19 patients occasionally suffer from akinetic mutism without disruption of the underlying frontal-subcortical circuits, as probably occurred in our second patient (59).

Therefore, comprehensive studies are needed to determine if delirium in COVID-19 patients represents a primary encephalopathy predicting virus invasion of the CNS, a secondary encephalopathy related to systemic inflammatory response, or even a condition associated with other factors.

The SARS-CoV-2 infection is associated with multiorgan involvement, including fever, cough, and shortness of breath, absent in our patients at admission and during the hospitalization. Differently, our patients did not develop the typical febrile response, as it occasionally occurs in older patients due to changes in thermoregulation and immune cell dysregulation (60). Finally, chest computed tomography (CT) examination advanced equipment for diagnosis (61), failed to demonstrate a pulmonary failure in all three patients.

The neurological examination and isolation of SARS-CoV-2 from cerebrospinal fluid (CSF) of affected patients might enable physicians to detect multiple neurological manifestations caused by the virus. Alternatively, a transcribrial spread of the virus to the brain is also supported by the presence of hyposmia/anosmia as being among the earliest symptoms commonly present. However, such a hypothesis needs to be further elucidated by isolating this virus from proximity to the olfactory bulb. We obtained no evidence for SARS-CoV-2 CNS direct involvement in our three patients since only the first patient received a lumbar puncture, which tested negative. Moreover, all patients presented neither anosmia nor ageusia. The sole evidence of the COVID-19 was given by the yielded positive results of the naso-oropharyngeal reverse transcription-polymerase chain reaction (RT-PCR) screening test.

Our study shows a few limitations. The first is the retrospective nature of the three cases’ clinical description and the formal diagnosis of mild cognitive impairment based on MMSE testing, which was probably used only as a first step in screening cognitive impairment and requiring more intensive diagnostic information. However, the MMSE has essential shortcomings, including lack of standardization and suitability for illiterate subjects, the considerable effect of socio-educational variables on results, and its limited effectiveness for detecting cognitive impairment (62). Thus, the sole anamnestic information and MMSE testing did not give us further information to characterize the complete cognitive impairment diagnosis.

The second is that we evaluated only in-hospital patients and could not report on outpatients with the same clinical features. The third is the limited sample with preexisting mild cognitive impairment, with only one patient having no psychiatric history and two patients with a psychiatric history.

Thus, our preliminary results should be consolidated in a larger cohort of patients to perform a systematic investigation designed to contribute to generalizable knowledge.

## Conclusion

COVID-19 complications involving psychiatric manifestations are yet to be clarified (63, 64). However, the scientists infer from current literature that patients with preexisting mental disorders appear more vulnerable to the risks of infection than the general population.

Our study revealed that delirium might represent a frequent outcome of SARS-CoV-2 infection in psychiatric patients, indicating that the virus might spread throughout the CNS without systemic symptoms.

Also, COVID-19 associated delirium appears to be the prodrome of a further mental disease episode in patients with a psychiatric history or the sole clinical manifestation of SARS-CoV-2 brain invasion in patients without a psychiatric history. In conclusion, the only feature the three patients share is the preexisting mild cognitive impairment that might have acted as a predisposing factor for COVID-19. Instead, psychiatric history and cognitive impairment might have played the role of predisposing factors for COVID-19 sequelae in delirium patients.

Therefore, clinicians should consider the possibility that SARS-CoV-2 presence in the brain might clinically manifest in the form of delirium and acute psychiatric sequelae, even without other systemic symptoms.

Mental health providers should be alert for the increased demand in the coming years due to the COVID-19 short- and long-term complications in psychiatric patients.

## Data Availability Statement

The raw data supporting the conclusions of this article will be made available by the authors, without undue reservation.

## Ethics Statement

Ethical review and approval was not required for the study on human participants in accordance with the local legislation and institutional requirements. The patients/participants provided their written informed consent to participate in this study.

## Author Contributions

MF, NC, and FC designed the study and interpreted the data and wrote the first draft of the manuscript. AR, AC, BA, and PC provided patients’ data and contributed to the first draft. MF and NC interpreted the data and wrote a revised version of the manuscript together with FC. ML provided constructive feedback, supervision, and a critical review of the manuscript. All authors contributed to the article and approved the submitted version.

## Conflict of Interest

The authors declare that the research was conducted in the absence of any commercial or financial relationships that could be construed as a potential conflict of interest.

## Publisher’s Note

All claims expressed in this article are solely those of the authors and do not necessarily represent those of their affiliated organizations, or those of the publisher, the editors and the reviewers. Any product that may be evaluated in this article, or claim that may be made by its manufacturer, is not guaranteed or endorsed by the publisher.
